# Application of hybrid improved temporal convolution network model in time series prediction of river water quality

**DOI:** 10.1038/s41598-023-38465-3

**Published:** 2023-07-12

**Authors:** Yankun Hu, Li Lyu, Ning Wang, Xiaolei Zhou, Meng Fang

**Affiliations:** 1 Shenyang Institute of Computing Technology, Chinese Academy of Sciences, Shenyang, 110168 Liaoning China; 2 University of Chinese Academy of Sciences, Beijing, 100049 China

**Keywords:** Computer science, Electrical and electronic engineering, Computational science

## Abstract

Time series prediction of river water quality is an important method to grasp the changes of river water quality and protect the river water environment. However, due to the time series data of river water quality have strong periodicity, seasonality and nonlinearity, which seriously affects the accuracy of river water quality prediction. In this paper, a new hybrid deep neural network model is proposed for river water quality prediction, which is integrated with Savitaky-Golay (SG) filter, STL time series decomposition method, Self-attention mechanism, and Temporal Convolutional Network (TCN). The SG filter can effectively remove the noise in the time series data of river water quality, and the STL technology can decompose the time series data into trend, seasonal and residual series. The decomposed trend series and residual series are input into the model combining the Self-attention mechanism and TCN respectively for training and prediction. In order to verify the proposed model, this study uses opensource water quality data and private water quality data to conduct experiments, and compares with other water quality prediction models. The experimental results show that our method achieves the best prediction results in the water quality data of two different rivers.

## Introduction

Rivers are the most common and basic source of water for many organisms, and play an important role in domestic water use, agricultural irrigation, and industrial development^1^. But due to the interaction between rivers and the surrounding environment, as well as the exchange of urban, industrial and agricultural pollutants along the way, the problem of river pollution is becoming more and more serious^2^. Water quality prediction and assessment are essential for the protection of human and environmental health and for effective and sustainable water resource management^3^. Although water quality testing can be performed using traditional techniques and methods, such methods are usually time-consuming, expensive, and less accurate. In recent years, the technology of water quality modeling and prediction based on machine learning and deep learning methods has been widely used and achieved better prediction results^4^.

Traditional models based on statistical analysis methods mainly include multiple linear regression (MLR), Autoregressive (AR), Autoregressive integrated Moving Average (ARIMA) and SARIMA models, etc.^5,6^. Jiang Wu et al.^7^ proposed a water quality prediction method combining ARIMA and clustering model, and taking the water quality monitoring data of a basin as a sample, the total phosphorus (TP) index of water quality was selected as the prediction object, and the water quality change in the basin was successfully predicted. Mohamed Elhag et al.^8^ used the adjusted ARIMA and SARIMA models to predict water quality parameters, and verified that the SARIMA model could effectively predict water quality parameters with seasonal characteristics. However, these models cannot capture the nonlinear characteristics in the data, resulting in low prediction accuracy and poor applicability of the model.

Machine learning regression analysis methods mainly include Support vector regression (SVR), Random forest regression (RFR), Bayesian Network (BN), Decision tree (DT), artificial neural network (ANN), BP neural network, etc.^9,10^. Theyazn H et al.^11^ carried out water quality prediction research based on machine learning algorithms such as Support vector Machine (SVM), K-Nearest Neighbor (K-NN) and Naive Bayes. S. Ayesha Jasmin et al.^12^ developed dissolved oxygen prediction models using three popular machine learning algorithms including Random Forest (RF), Adaboost and deep neural networks. Such models can deal with nonlinear features in data to a certain extent, but the prediction ability of the model is limited.

The application of deep learning models in water quality prediction mainly focuses on recurrent neural network (RNN), LSTM, Bi-LSTM, GRU, CNN models, etc. With the development of technology, more and more hybrid deep learning models have been developed and applied, such as CNN-LSTM, Attention-Bi-LSTM model, etc.^13^. Sakshi Khullar et al.^14^ used the deep learning Bi-LSTM method to predict the water quality of the Yamuna River in India. Yurong Yang et al.^15^ proposed a water quality prediction model combining convolutional neural network (CNN), Long Short-Term memory network (LSTM) and Attention mechanism, which has a strong ability to solve nonlinear time series prediction problems. Although the feature extraction ability and long-term memory ability of the model are further improved by integrating with CNN, Attention and other models, the problems of gradient disappearance and long training time are still not effectively solved.

In order to solve the problems existing in the above models and improve the accuracy of water quality prediction, we first used the SG filter^16^ to eliminate the noise in the original water quality data, and then decomposed the water quality data into trend, seasonal and residual series based on the STL time series decomposition method^17^. The decomposed trend and residual series will be separately used for model training and prediction, so as to better realize the extraction of features. Bai et al.^18^. proposed the TCN model in 2018, which introduces causal convolution, dilated convolution, and residual blocks. Compared with the RNN model, TCN does not have the gradient vanishing problem and has a longer memory capacity; TCN supports parallel computation, which means that each weight in each layer can be updated simultaneously at each time step, significantly improving the model computational efficiency^19^. The TCN model integrates the feature convolution processing capability of the CNN model and the time series information mining capability of the recurrent neural network, and has been widely studied and applied in a variety of time series forecasting problems such as load forecasting^20^ and wind speed forecasting^21^. Current research has demonstrated the superiority of TCN models over traditional machine learning algorithms as well as LSTM, GRU and other models on a variety of tasks and datasets. Therefore, in our model, TCN is used to extract the long-term dependencies in the sequences. At the same time, in order to further improve the performance of TCN model and solve the problem of local information loss of TCN model, we combine the Self-attention mechanism with the TCN model^22^, so that the model can pay more attention to the features that contribute more to the output, so as to have stronger feature extraction ability. In this paper, our contributions can be summarized as follows:

1. We use the SG filter to smooth the time series of river water quality, thereby eliminating the strong noise in the data and enhancing the availability of the data.
2. Based on the characteristics of river water quality data, we use STL decomposition technology to decompose the original water quality data into three sub-series: trend, seasonality and residual, which better retains the seasonality in the series and improves the prediction accuracy of the model.
3. The TCN model is improved, and the Self-attention mechanism is added to the residual block structure of TCN to further improve the feature extraction ability of the model.
4. The proposed river water quality prediction model is tested in two real river water quality data sets, and compared with other commonly used water quality prediction models, which verifies that our method can achieve the best prediction effect.

## Methodology

**Figure 1:**
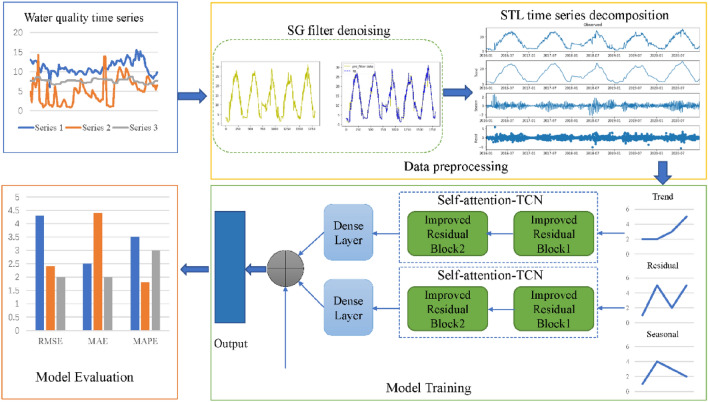
Overall structure and workflow of the model.

The overall structure and workflow of the model are shown in Fig. 1. In our proposed model structure, the collected water quality time series are firstly smoothed by the SG filter, so as to eliminate the strong noise in the data. Then, the smoothed data are decomposed by STL decomposition technology to obtain three sub-series: seasonality, trend and residual. Then, the trend series and residual series obtained by decomposition are input into the improved TCN model for feature extraction, and the trend series prediction value, residual series prediction value and retained seasonal series are fused to obtain the final prediction result of the model. Finally, the predicted value was compared with the real value, and the model was evaluated by a series of evaluation criteria.

## Savitzky-Golay (SG) filter

Smoothing and denoising the original time series data can effectively improve the accuracy of prediction^23^. Therefore, in this study, we used the SG filter to remove the noise in the original water quality time series. The SG filter is a filtering method based on the local polynomial least squares fitting in the time domain. The biggest feature of this filter is that it can ensure the shape and width of the signal are unchanged while filtering out the noise.

A subsequence $x$ with window size $w=2m+1$ can be expressed as:

1 $$\left\{{x}_{s-m},. . . ,{x}_{s}, . . . ,{x}_{s+m}\right\}, s\in \left[m+1, T-m\right].$$

The polynomial $p\left(i\right)$ of order $R$ used to fit the data points in the window is defined as follows:

2 $$p\left(i\right)=\sum_{v=0}^{R}{a}_{v}{i}^{v}, i\in \left[-m,m\right],$$

where ${a}_{v}$ denotes the v-th coefficient of the SG filter.

Then, use the least squares method to minimize the error $\epsilon$.

3 $$\epsilon =\sum_{i=-m}^{m}{(p\left(i\right)-{x}_{s+i})}^{2}.$$

Then we can find the best fit $p\left(0\right)$ of the window center point ${x}_{s}$ by computing ${a}_{0}$. By sliding the window, each point in the series $x$ will become the center point in the window until all the values in the series are smoothed. Finally, we will get the smoothed sequence ${x}{\prime}$.

### STL time series decomposition method

In order to better extract the trend characteristics and nonlinear characteristics of river water quality time series, and retain the seasonal trend characteristics of the series, we used STL decomposition technology to decompose the original water quality time series. Seasonal and Trend decomposition using Loess (STL) is a very general and robust decomposition method for time series, where Loess is a method for estimating nonlinear relationships. STL aims to decompose the time series data ${Y}_{v}$ at a certain time into trend (${T}_{v}$), season (${S}_{v}$) and residual (${R}_{v}$), denoted as ${Y}_{v}={T}_{v}+{S}_{v}+{R}_{v}$^24^. The algorithm consists of an outer loop and an inner loop. The outer loop is mainly used to assign a robust weight to each data point through the residual, so as to reduce the influence of outliers. The inner loop is nested in the outer loop and mainly does trend fitting and periodic component calculation. The process of the k-th epoch in the inner loop is as follows^25^:

1. Detrending. Remove the trending component from the original series, and get ${Y}_{v}-{T}_{v}^{(k)}$.
2. Cycle-subseries smoothing. Each cyclic-subseries obtained from step 1 is smoothed by Loess, resulting in a preliminary seasonal series denoted as ${C}_{v}^{(k+1)}$.
3. Low-Pass Filtering. The sequence ${C}_{v}^{(k+1)}$ obtained from step 2 is processed by moving average and Loess regression to obtain the result sequence ${L}_{v}^{(k+1)}$, which is equivalent to extracting the low-pass of the periodic subsequence.
4. Detrending of Smoothed Cycle-subseries. Calculate seasonal trends ${S}_{v}^{(k+1)}={C}_{v}^{(k+1)}-{L}_{v}^{(k+1)}$.
5. Deseasonalizing. Subtract the periodic component, ${Y}_{v}-{S}_{v}^{(k+1)}$.
6. Trend Smoothing. Loess regression is performed on the cycled series obtained in step 5 to obtain the trend component ${T}_{v}^{(k+1)}$.

When the inner loop reaches the accuracy requirement, the outer loop starts, and the residual component ${R}_{v}^{(k+1)}$ is calculated by the estimated trend and seasonal components in the outer loop. The calculation method is as follows:

4 $${R}_{v}^{(k+1)}={Y}_{v}-{T}_{v}^{\left(k+1\right)}-{S}_{v}^{(k+1)}.$$

### Improved TCN model

**Figure 2:**
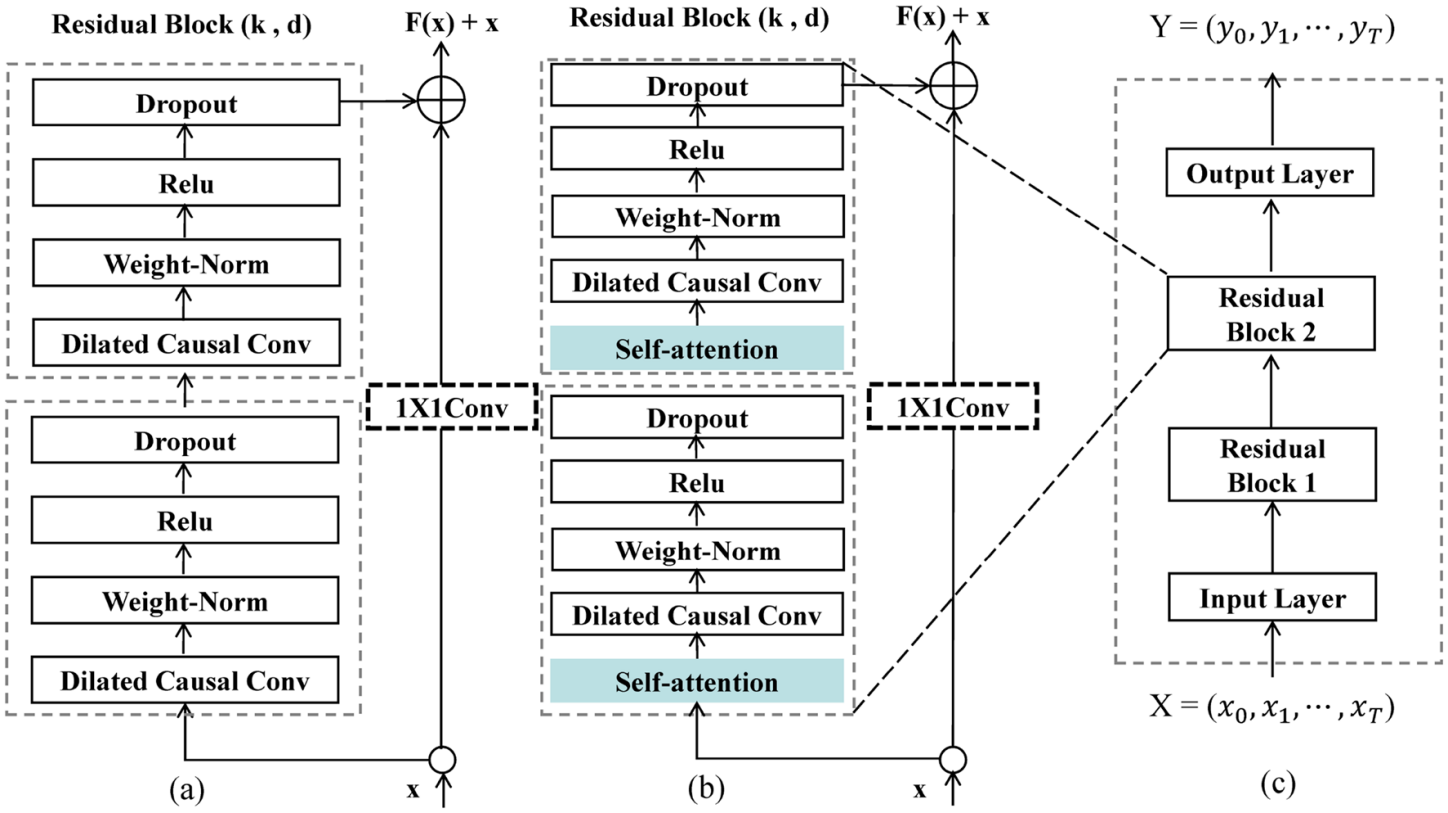
(**a**) The Standard TCN residual block structure, (**b**) The improved TCN residual block structure, (**c**) The TCN network structure we adopted.

Temporal convolutional network (TCN) is based on the traditional one-dimensional fully convolutional neural network model, and combines causal convolution, dilated convolution and residual block structure, so that the model has the ability to extract features from time series and achieve prediction, and can effectively solve the performance degradation problem of deep networks in the process of network training^26^. Figure 2a shows the standard TCN residual block structure, including dilated causal convolution, Weight Norm, ReLU activation function, and Dropout regularization. The dilated causal convolution is used for feature extraction, the Weight Norm can regulate the input of the hidden layer to counteract the gradient explosion problem of the network, the ReLU activation function introduces nonlinearity into the model, and the Dropout regularization operation can randomly drop neurons according to a certain probability, so as to prevent overfitting and accelerate the model training speed. If $x$ is the input of the residual block, the output of the residual block $o$ can be expressed as follows:

5 $$o=Activation\left(x+F\left(x\right)\right),$$

where $Activation$ is the activation function and $F(x)$ is the residual. Since the residual $F(x)$ is not 0 in practice, the stacked layers in the deep learning network can always learn new features, so the learning performance of the deep network will not degrade.

**Figure 3:**
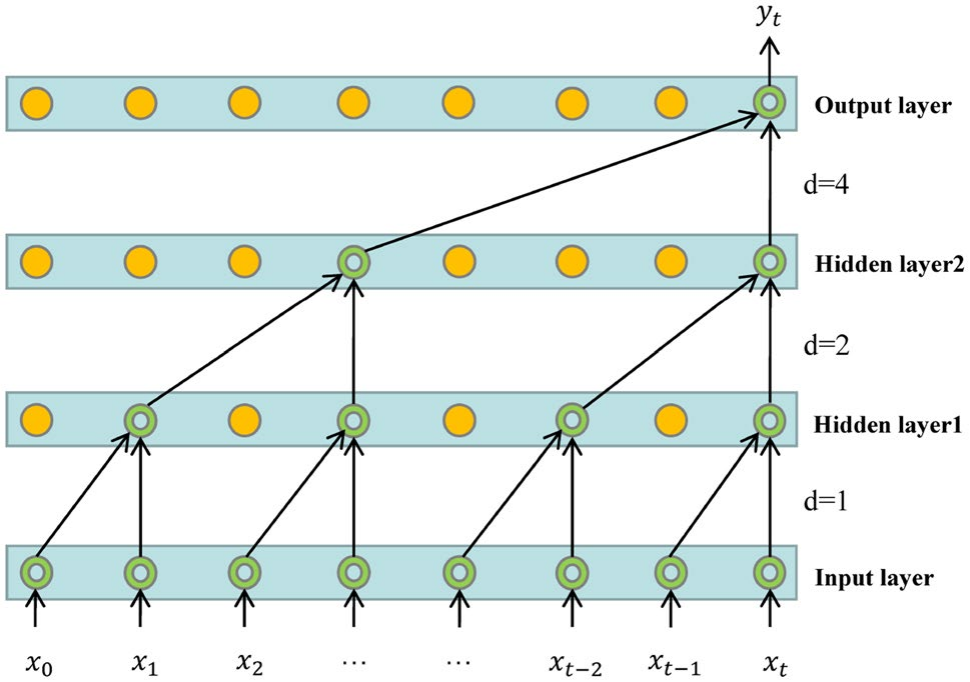
Dilated causal convolutions with dilation factors d = 1,2,4 and kernels k = 2.

Casual Convolutions were originally proposed in the WaveNets network. Since the traditional CNN model cannot directly deal with the sequence problem, causal convolution can abstract the sequence into according to ${x}_{1}, {x}_{2},. . .,{x}_{t}$ and ${y}_{1}, {y}_{2},. . .,{y}_{t-1}$ to predict ${y}_{t}$ and make ${y}_{t}$ close to the actual value. Compared with recurrent neural networks (RNNS), models using causal convolutions do not use recurrent connections, thus allowing time series data to be input in parallel, which makes the network training faster and has greater advantages when the amount of data is large^27^. However, in order to expand the receptive field of neural network neurons in standard causal convolution, many network layers need to be stacked or large convolution kernels need to be used. In order to solve the problem of limited receptive field of standard causal Convolution, TCN combines Dilated convolution with causal convolution, and uses Dilated Casual Convolution (DCC) to increase the receptive field of neurons without causing a significant increase in computational cost^28^. The one-dimensional dilated causal convolution operation is expressed as follows:

6 $$F\left(s\right)= \sum_{i=0}^{k-1}f(i){x}_{s-di},$$

where $x$ is the input sequence, $f(i)$ is the filter, also known as the convolution kernel, $d$ is the dilation factor, $k$ is the size of the convolution kernel, $s-di$ ensures that only past inputs can be convolved. Figure 3 shows the dilated causal convolution structure with dilation factors $d=\mathrm{1,2},4$ as well as the convolution kernel $k=2$.

**Figure 4:**
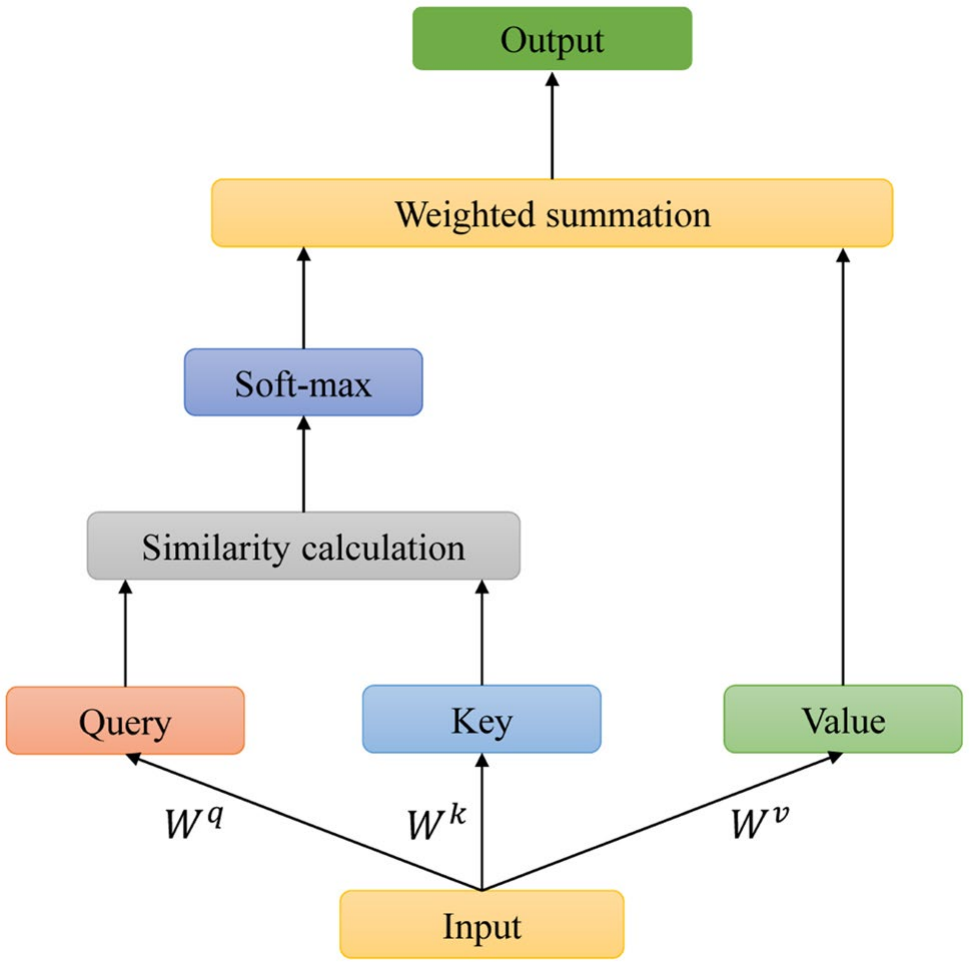
Self-attention structure diagram.

Attention mechanism is mainly a simulation of the form of human brain attention allocation, and its essence is to assign weights to different features in the hidden layer, so as to highlight the influence of important features^29^. The Self-attention mechanism is an improvement of the attention mechanism, which aims to capture the internal correlation of the data, so as to further improve the prediction ability of the model^30^. The structure of self-attention mechanism is shown in Fig. 4, it consists of Q(Query), K(Key), and V(Value) vectors, which are obtained by multiplying the input data by three matrices ${W}_{q}$, ${W}_{k}$, and ${W}_{v}$. In our proposed model, ${W}_{q}$, ${W}_{k}$, and ${W}_{v}$ are the transformations of water quality data, and the attention matrix used to determine the feature attention can be calculated by Eq. (7).

7 $$attention\left(Q,K,V\right)=softmax\left(\frac{Q{K}^{T}}{\sqrt{{d}_{K}}}\right),$$

where* T* refers to the matrix transpose and ${d}_{K}$ refers to the dimension of *K*.

We add Self-attention before each convolutional unit in the TCN residual block structure, and the improved TCN residual block structure is shown in Fig. 2b. Based on the above improved residual block structure, the basic structure of the deep TCN model adopted in this paper is shown in Fig. 2c. The model consists of an input layer, an improved residual block, and an output layer. The input layer mainly receives the decomposed water quality time series data. Two stacked residual block structures were used to increase the depth of the model and make the model training more adequate. The output layer is a fully connected layer, which receives the output vector of the TCN model and calculates the predicted value.

## Empirical analysis

### Dataset and model evaluation criteria

The opensource dataset uses the environmental estuary water quality monitoring data of the Burnett River in Queensland, Australia, which can be obtained from the Queensland Government Open Data portal. The raw data contains measurements of temperature, PH, dissolved oxygen, electrical conductivity, turbidity, and chlorophyll concentration in the water measured at 30-min intervals from 2014 to 2018^31^. Our experiment was mainly to predict the dissolved oxygen concentration value, so we extracted the dissolved oxygen concentration data from the original data separately and resampled the data on a daily basis. However, there were missing data in the data after resampling, and we used the mean value to fill the missing data.

The private data set used in our experiment is from the Liao River automatic monitoring Station in China. The data set contains the dissolved oxygen concentration measurements recorded every 4 h from January 1, 2016 to August 31, 2022. Similarly, the original data set was resampled on a daily basis, and the missing values in the data were filled with the mean value.

To effectively evaluate our model, Root Mean Square Error (RMSE), Mean Absolute Error (MAE), and Mean Absolute Percentage Error (MAPE) were used to measure the difference between the predicted value and the true value. The smaller the error value is, the closer the predicted value is to the true value, and the higher the prediction accuracy of the model^32^. The calculation formula is as follows:

8 $$RMSE=\sqrt{\frac{1}{n}\sum_{t=1}^{n}{\left({y}_{t}-{\widehat{y}}_{t}\right)}^{2}},$$

9 $$MAE=\frac{1}{n}\sum_{t=1}^{n}\left|{y}_{t}-{\widehat{y}}_{t}\right|,$$

10 $$MAPE=100\times \frac{1}{n}\sum_{t=1}^{n}\left|\frac{{\widehat{y}}_{t}-{y}_{t}}{{y}_{t}}\right|,$$

where n represents the sequence length, ${y}_{t}$ and ${\widehat{y}}_{t}$ represent the true and predicted values of the model at time t, respectively.

### Data smoothing

We will use the SG filter to smooth the noise present in the water quality data. Two important parameters in the SG filter determine the ability of the filter to remove noisy data, which are the window size M and the K value. It should be noted that the value of M must be a positive odd integer. If the value of M is set too large, it will remove the temporal features in the sequence, and too small, it will not be used to reduce the noise in the sequence. The value of K indicates that the points in the window are fitted by a k-order polynomial. If the value of K is too large, it will lead to least squares overfitting, and if it is too small, it will lead to underfitting. Therefore, in order to get a better SG filter, we set a variety of different parameter combinations, and determine the best parameter combination according to the RMSE, and the results are shown in Table 1.

**Table 1:** Results of different parameter combinations of SG filter.

Window size (M)	K value size	Burnett River (RMSE)	Liao River (RMSE)
5	3	0.122	0.336
7	3	0.162	0.437
7	5	0.109	0.300
9	3	0.205	0.514
9	5	0.138	0.385
11	7	0.126	0.353

According to Table 1, when the window size M is set to 7 and the K value is set to 5, the SG filter obtains the smallest RMSE value in both datasets, so we determine the best parameter combination M = 7 and K = 5 in this experiment.

### STL data decomposition

**Figure 5:**
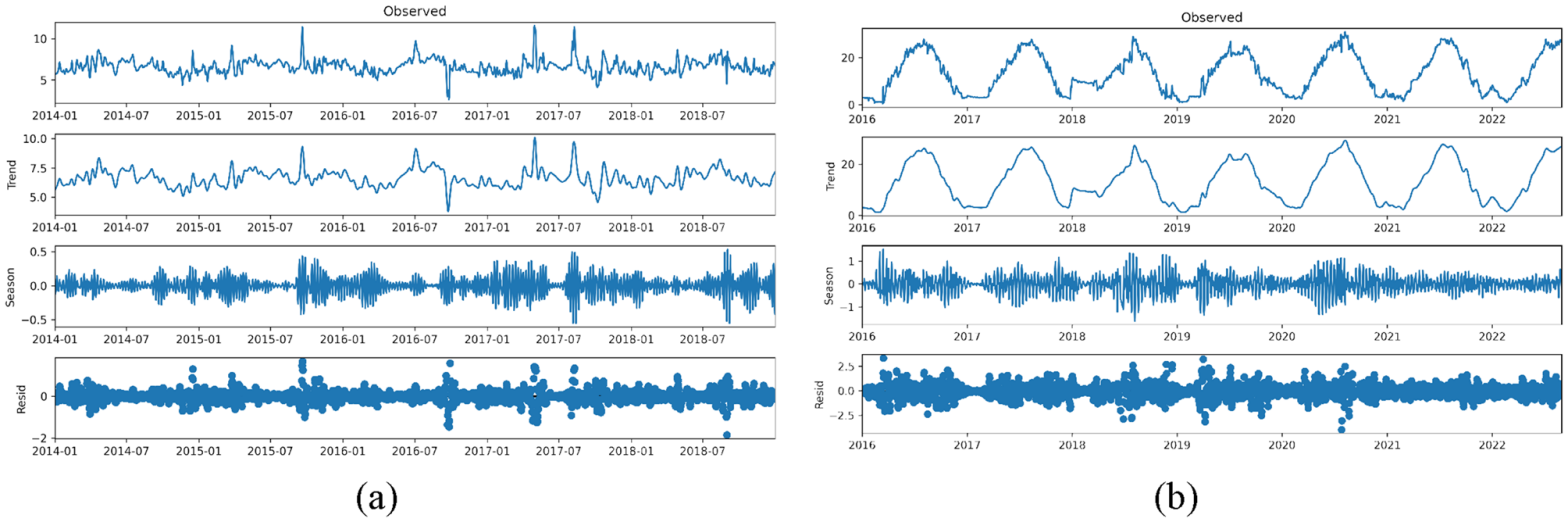
(**a**) Decomposition results of the water quality data of the Burnett River, (**b**) Decomposition results of the water quality data of the Liao River.

Now, we will decompose the smoothed data using the STL technique to decompose the original time series into trend, seasonal, and residual series. In the experiment, the decomposition of time series is realized by calling the STL method in the statsmodels class library. The decomposition results of the water quality data of Burnett River are shown in Fig. 5a, and the decomposition results of the water quality data of Liao River are shown in Fig. 5b.

**Figure 6:**
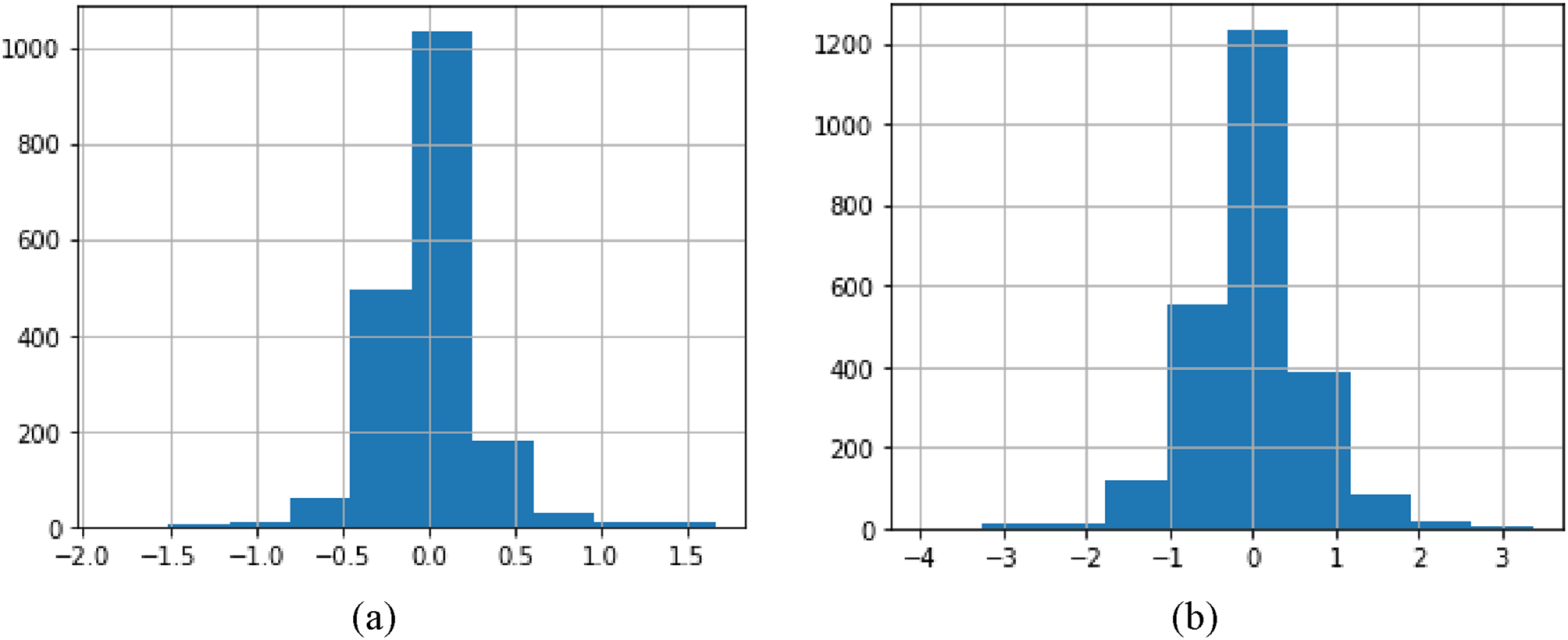
(**a**) Residual distribution of Burnett River water quality data, (**b**) Residual distribution of Liao River water quality data.

In order to verify that we used the correct time series decomposition method, we further observed the residual distribution and the mean value of the time series. According to Fig. 6a and b, the residual values of the two water quality data show an approximate normal distribution with the mean value of 0, so it can be shown that the decomposition method we adopted is correct.

### Model training and prediction

Next, we will train our proposed model based on the trend series and residual series obtained after decomposition and make predictions. We take 80% of the data set as the training set, 20% as the test set, and the data set is normalized by the min–max normalization method. In the experiment, we take the water quality data of the first 7 days as the input of the model, and the output is the predicted value of the next day, that is, the sliding window size is 7, and the prediction step size is 1.

In order to prove the prediction ability of the proposed model, we set up baseline models to compare with our model, including Support Vector Regression (SVR), Long Short-Term Memory network (LSTM), Temporal Convolutional Network (TCN) and TCN + Self-attention model. All models were tested in the same environment, and the same evaluation criteria were adopted for comparative analysis. The main parameter Settings of each model in the test are shown in Table 2.

**Table 2:** Parameter Settings of each model.

Model	Parameters
SVR	C = 10, kernel = ’rbf’, epsilon = 0.1
LSTM	Units = 32, num_layer = 2, dense = 1
TCN	nb_filters = 64, kernel_size = 2, nb_stacks = 2
TCN + Self-attention	nb_filters = 64, kernel_size = 2, nb_stacks = 2, units = 32
Our model	M = 7, k = 5, nb_filters = 64, kernel_size = 2, nb_stacks = 2, units = 32

## Results analysis

**Figure 7:**
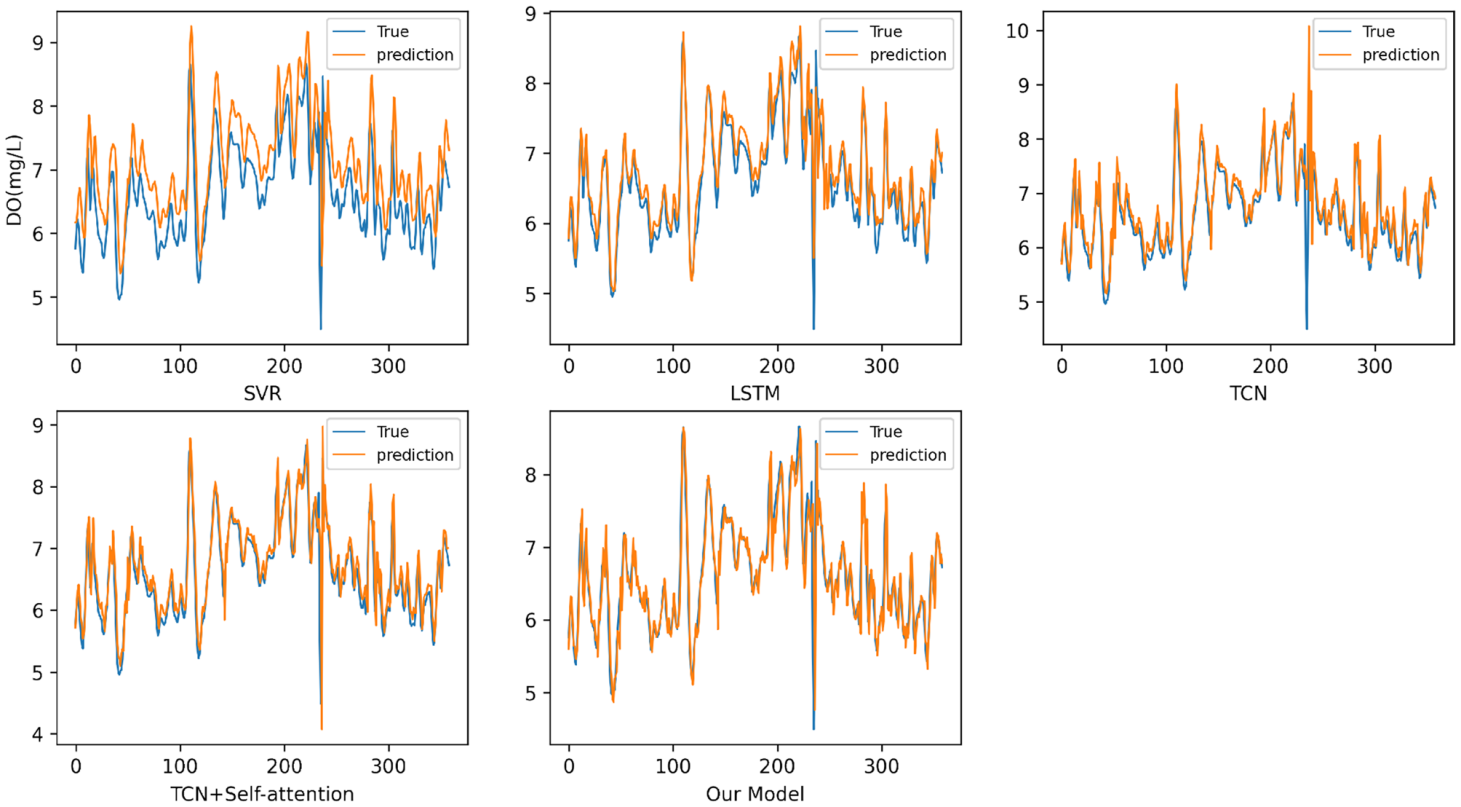
Prediction curves of water quality data of the Burnett River on various models.

The prediction results of the Burnett River water quality data on various prediction models are shown in Table 3. According to Table 3, the results of our proposed model are the best among various evaluation indicators, which proves that the model has higher prediction accuracy and better prediction ability. In order to show the gap between the predicted results of various models and the real monitoring values more intuitively, we show the predicted values and the real values through the curve. The comparison of the prediction result curves of various prediction models on this data set is shown in Fig. 7.

**Table 3:** Prediction results of water quality data of the Burnett River.

Model	RMSE	MAE	MAPE
SVR	0.6818	0.5409	7.7094
LSTM	0.5729	0.3234	5.7459
TCN	0.4132	0.3018	4.2751
TCN + Self-attention	0.3042	0.2353	3.9408
Our model	0.2439	0.1901	2.9189

**Figure 8:**
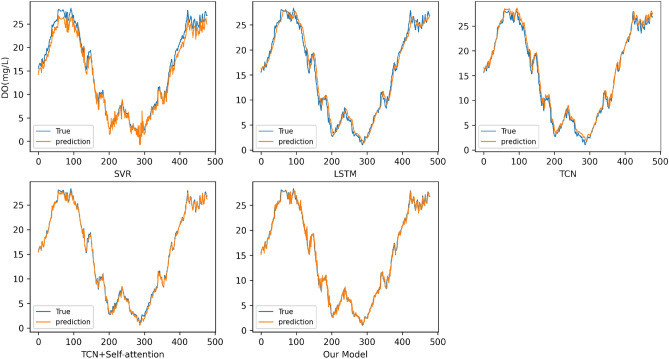
Prediction curves of water quality data of the Liao River on various models.

Table 4 shows the prediction results of various comparison models on the Liao River water quality data. According to Table 4, The prediction result of SVR model is the worst, the prediction result of TCN model is better than LSTM, and the prediction ability of TCN model with the addition of Self-attention is greatly improved. The prediction results of our model on this data set are still the best, indicating that our model has stronger generalization ability, and the model has a good application prospect in the field of river water quality prediction. The comparison of the prediction curves of various prediction models on this data set is shown in Fig. 8.

**Table 4:** Prediction results of water quality data of the Liao River.

Model	RMSE	MAE	MAPE
SVR	1.2836	1.0863	11.3279
LSTM	0.9274	0.7739	9.2364
TCN	0.7432	0.5583	6.3728
TCN + Self-attention	0.6908	0.4624	5.1906
Our model	0.4082	0.3299	4.5664

## Conclusion

Water quality prediction models are very important for water environment management. However, due to the complex and variable characteristics of water environment and the periodicity and nonlinearity of water quality data, it is a great challenge to accurately predict water quality. Therefore, in this study, we propose a hybrid improved temporal convolutional network model, which combines SG filter, STL time series decomposition method, TCN model and Self-attention mechanism. We use the SG filter to remove the noise in the water quality data, and use the STL decomposition method to decompose the water data into trend, seasonal and residual series. We improve the residual block structure of the standard TCN model and add the Self-attention mechanism to improve the prediction ability of the TCN model. Finally, we use opensource water quality data and private water quality data to conduct experimental verification. The results show that our proposed model has higher prediction accuracy than several other commonly used benchmark models. The water quality prediction model proposed in our present study can predict the water quality changes of rivers in the future period more accurately, thus helping managers to make early warnings of water pollution and take necessary measures in advance to deal with water pollution problems and better protect the water environment.

The limitation of this study is that our model only takes the historical water quality data as the model input, and does not consider the influence of other relevant features on the data. Therefore, in the next step, we will analyze the influence of different external factors on water quality changes and consider more features as the input of the model, so as to further improve the practicability of the model in real scenarios. In addition, we will continue to use water quality data of different rivers and different pollutants to evaluate the prediction ability of the model, further optimize the model structure, explore larger prediction steps, and carry out in-depth research on long-term series prediction.

## Data Availability

The data sets used and/or analyzed during the current study are available from the corresponding author on reasonable request.
